# Biologically Driven Tooth Regeneration: A Scoping Review of Stem Cell-Based Approaches

**DOI:** 10.7759/cureus.106495

**Published:** 2026-04-05

**Authors:** Niti Singh, Daniel E. E Moore Jr, Adarsh Keshari

**Affiliations:** 1 Biology, Great Valley High School, Malvern, USA; 2 Prosthodontics, Visionary Dental, West Chester, USA; 3 Clinical Research and Capacity Building, APAR Health, Gurugram, IND

**Keywords:** dental stem cells, dental tissue engineering, regenerative dentistry, stem-cell therapy, tooth regeneration

## Abstract

Regenerative dentistry is an emerging branch of medicine that focuses on restoring biological tooth structures using stem cells, biomimetic scaffolds, and signaling factors, rather than relying on traditional man-made materials. Current treatments, such as fillings and crowns, can rebuild lost tooth structures but cannot replicate the structure and function of dentin-pulp tissues. Recent preclinical and early human studies have explored various stem-cell sources, including dental pulp stem cells (DPSCs), stem cells from exfoliated deciduous teeth (SHED), and periodontal ligament stem cells (PDLSCs). Among these, DPSCs and SHED have been the most extensively investigated. Most studies have shown that, when combined with pro-angiogenic factors such as vascular endothelial growth factors (VEGFs) and fibroblast growth factors (FGFs), collagen and hydrogel scaffolds are effective in regenerative dentistry.

These studies have demonstrated successful tissue formation, mineralization, and vascularization in animal models and early human trials. However, considerable variability exists among studies due to differences in experimental design, stem-cell sources, scaffold materials, and growth-factor use, leading to inconsistent outcomes. Despite these advances, the literature remains fragmented and largely preclinical. Only a limited number of human studies are available, and variability among studies remains substantial. Existing reviews often focus on a single component, such as a specific stem-cell type or scaffold property, rather than integrated regenerative systems. This scoping review, conducted in accordance with the Preferred Reporting Items for Systematic Reviews and Meta-Analyses Extension for Scoping Reviews (PRISMA-ScR) guidelines, synthesizes available preclinical and early clinical evidence and identifies combinations of stem cells, scaffolds, and signaling molecules with the greatest potential for predictable dental tissue regeneration.

## Introduction and background

Regenerative medicine is one of the most dynamic areas in modern biomedical science, and dentistry is rapidly becoming an integral part of this revolution. Recent research has highlighted that dental stem cells, biomaterials, and tissue engineering strategies are increasingly central to dental tissue repair and regeneration [1]. Traditional restorative approaches, including fillings, crowns, and dental implants, are widely used to restore or replace damaged tooth structures; however, these methods do not restore the biological and functional properties of living dental tissues, often necessitating long-term maintenance and occasional replacement [2].

Regenerative dentistry has therefore emerged as a promising field aimed at restoring missing or damaged dental tissues through biologically driven approaches. This discipline seeks to harness the body’s intrinsic repair mechanisms using stem cells, biomaterials, and bioactive signaling molecules [3]. In this regenerative framework, stem cells serve as a source of progenitor cells capable of differentiation, biomaterials act as scaffolds that mimic the three-dimensional structure of the extracellular matrix, and signaling molecules regulate cellular proliferation, differentiation, and vascularization [4]. Growth factors such as bone morphogenetic proteins (BMPs), fibroblast growth factors (FGFs), and vascular endothelial growth factors (VEGFs) play critical roles in modulating cellular behavior and promoting tissue regeneration.

Over the past two decades, numerous studies have investigated various stem cell sources for dental tissue engineering. Dental pulp stem cells (DPSCs) were first identified as multipotent stem cells capable of generating dentin-like structures in vivo [5]. Subsequently, stem cells derived from human exfoliated deciduous teeth (SHED) demonstrated strong proliferative and regenerative potential for dental tissue formation [6]. These discoveries significantly advanced regenerative dentistry and highlighted the potential of dental-derived stem cells for tissue engineering applications.

Pluripotent stem cells, including embryonic stem cells (ESCs) and induced pluripotent stem cells (iPSCs), also demonstrate significant differentiation potential toward odontogenic lineages [1]. However, their clinical application remains limited due to ethical concerns associated with ESCs and the potential tumorigenic risk associated with both ESCs and iPSCs. In contrast, DPSCs and SHED have shown promising regenerative potential in dentin-pulp complex formation in experimental models, particularly when combined with pro-angiogenic factors such as VEGF and FGF [3]. Additionally, periodontal ligament stem cells (PDLSCs) and stem cells from the apical papilla (SCAP) have demonstrated the ability to regenerate periodontal structures by differentiating into cementoblast-like and periodontal ligament cells [7]. Preclinical investigations further suggest that combining dental stem cells with biomimetic scaffolds may improve tissue thickness, mineralization, and cellular survival during regenerative processes [4]. However, reported outcomes remain heterogeneous due to variations in scaffold composition, growth factor concentrations, differentiation protocols, and experimental conditions.

Most available evidence has been derived from in vitro studies or animal models, while human clinical investigations remain limited and largely at early feasibility stages [3]. Several uncertainties persist regarding the long-term stability of regenerated tissues, functional integration, neural innervation, and regulatory considerations associated with stem-cell-based therapies. In this context, a comprehensive mapping of available evidence is necessary to better understand the current status and future potential of stem-cell-based regenerative dentistry. Previous reviews in this field have largely been narrative and focused on individual components of the regenerative system, such as specific stem-cell populations or scaffold materials, rather than examining the integrated regenerative framework.

Therefore, the present scoping review, conducted in accordance with the Preferred Reporting Items for Systematic Reviews and Meta-Analyses Extension for Scoping Reviews (PRISMA-ScR) guidelines, aims to systematically identify and map the nature and extent of available preclinical and early clinical evidence on stem-cell-based regenerative approaches in dentistry. Using the Population-Intervention-Comparator-Outcome (PICO) framework, this review evaluates regenerative strategies involving stem cells alone or in combination with biomaterials and signaling molecules compared with conventional non-regenerative approaches, with respect to tissue regeneration, vascularization, integration, and long-term stability.

## Review

Methodology

The inclusion criteria for the studies were that they focus on regenerative dental therapies, including stem-cell-based regenerative approaches, alone or in combination with biomimetic scaffolds, as well as signaling molecules. Eligible studies included original preclinical investigations, early human clinical studies, and review articles relevant to regenerative dentistry. In accordance with the scoping review approach, narrative and systematic review articles that synthesized original preclinical or early clinical data pertinent to stem-cell-based regenerative dentistry were also included to identify research themes in addition to specific studies.

To ensure standardization, only studies specifying the source of stem cells (e.g., DPSCs, SHED, PDLSCs, SCAPs, iPSCs), along with the type of scaffold or growth factor, were included. Studies in which only synthetic scaffolds were used, as well as non-dental regenerative therapies, were excluded (Table 1). Studies investigating platelet-rich plasma (PRP) or acellular scaffolds alone were excluded as primary evidence; however, PRP-focused reviews were included for contextual framing and background discussion. As this study utilized only publicly available data, ethical approval was not required.

**Table 1:** Eligibility criteria for study inclusion in the scoping review PRP: platelet-rich plasma

Category	Inclusion criteria	Exclusion criteria
Population	Human or animal dental tissues requiring regeneration	Non-dental issues
Intervention	Stem-cell-based regenerative therapies	PRP, acellular scaffolds, synthetic-only materials
Outcomes	Dentin, pulp, periodontal, and enamel-like regeneration	Non-regenerative outcomes
Study type	In vitro, animal, and early human trials	Editorials, case reports
Language	English	Non-English
Publication date	2010–2025	Outside the year range

The literature search was conducted via the following databases: PubMed, Scopus, Web of Science, and Cochrane Library. Additionally, literature was obtained through professional organizations for biomaterials and regenerative dentistry. The literature search was based on the following keywords and their combinations: “regenerative dentistry,” “dental tissue engineering,” “stem cells,” “dental pulp stem cells,” “SHED,” “PDLSCs,” “SCAP,” “iPSCs,” “scaffold,” “biomaterial,” “hydrogel,” “nanofiber,” “growth factor,” “VEGF,” “FGF,” and “BMP.” These terms were combined using the following keywords: (“regenerative dentistry” OR “dental tissue engineering”) AND (“stem cells” OR “dental pulp stem cells” OR SHED OR PDLSCs OR SCAP OR iPSCs) AND (“scaffold” OR “biomaterial” OR hydrogel OR nanofiber) AND (“growth factor” OR VEGF OR FGF OR BMP). The literature was also filtered according to language, mammalian and human models, and the publication year range of 2010 to 2025. Literature management and screening were conducted independently by the reviewers with the help of tools such as Rayyan and others.

Results and discussion

**Figure 1:**
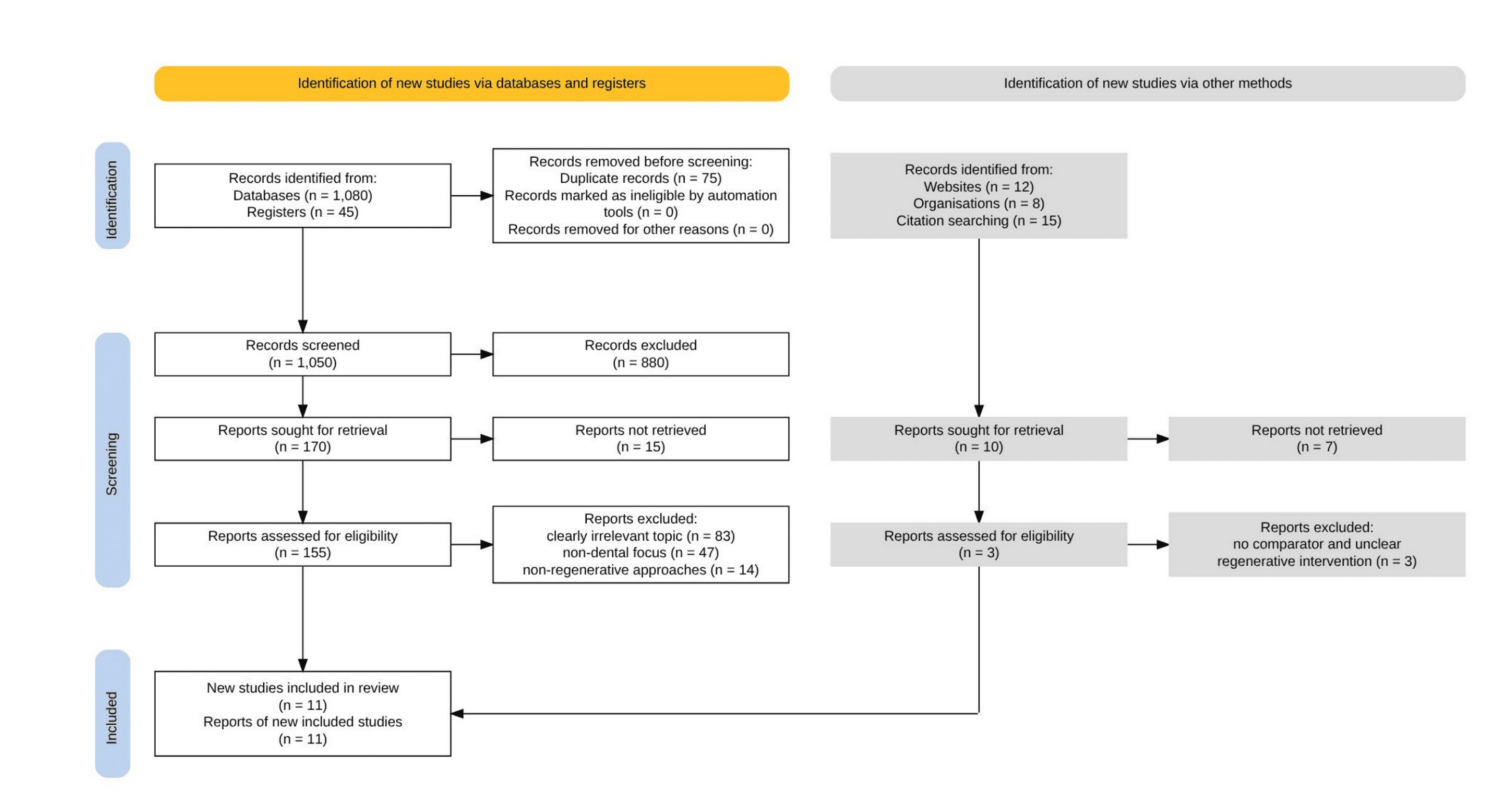
PRISMA-ScR flow diagram showing the study selection process for stem-cell-based regenerative dentistry review PRISMA-ScR: Preferred Reporting Items for Systematic Reviews and Meta-Analyses Extension for Scoping Reviews

A total of 1,080 records were identified through database searching. After the removal of 75 duplicate records, 1,005 articles remained for title and abstract screening. During this stage, 850 records were excluded due to their lack of relevance to regenerative dentistry, a non-dental focus, or the absence of stem-cell-based regenerative approaches. Consequently, 155 full-text articles were retrieved and assessed for eligibility. Of these, 144 articles were excluded based on the predefined inclusion and exclusion criteria, including studies focusing solely on PRP, acellular scaffolds, or non-regenerative dental interventions. Ultimately, 11 studies met the eligibility criteria and were included in the final scoping review. The study selection process is illustrated in the PRISMA-ScR flow diagram (Figure 1) [8].

The studies were systematically classified according to the type of stem cells utilized, the strategy of scaffold-based versus scaffold-free approaches, and the application of signaling molecules or growth factors [9]. This allowed for a qualitative analysis of the various regenerative strategies. The consistency of regenerative outcomes, including dentin-pulp tissue formation, vascularization, mineralization, and tissue integration, was also assessed across groups. Differences among the studies in terms of experimental conditions (in vitro, animal, and human studies), the materials used, and the provision of angiogenic conditions were taken into account when interpreting the findings. This screening and selection process was conducted independently by two researchers. As recommended by the PRISMA-ScR checklist, quantitative analysis was not conducted. Instead, the methodological quality of the studies was used to confirm the robustness of the narrative findings (Table 2).

**Table 2:** Characteristics of the studies included in the scoping review

Study	Primary outcomes reported	Secondary outcomes reported	Outcomes missing/not reported
Baena et al. [10]	The review summarizes dental stem cell differentiation, organoid formation, and lineage-specific regenerative capacity. It emphasizes their ability to generate odontogenic and osteogenic markers	It also discusses angiogenesis and mineralization markers associated with dental stem cell-mediated repair. Additional pathways related to signaling and regeneration are highlighted	No functional tooth regeneration studies were presented. Long-term clinical evidence was also missing
Amrollahi et al. [11]	The study outlines biomaterial advancements in dentin, pulp, and bone regeneration. It reviews evidence of mineral deposition and scaffold-supported tissue formation	It includes a discussion of how materials support integration and biological signaling. Translational considerations for clinical use are briefly covered	No consistent clinical endpoints were reported. No standardized regeneration outcome measures were provided
Xiao and Nasu [12]	This review describes stem cell-based regeneration mechanisms in dentistry. It explains differentiation pathways relevant to pulp, bone, and periodontal restoration	It also explores how dental regenerative strategies relate to broader regenerative medicine. High-level comparisons among cell types are included	No primary experimental outcomes were provided. The article lacked a quantitative measurement of regenerative success
Bansal and Jain [13]	The article reviews multiple dental stem cell types and their differentiation potential. It reports their capacity to form mineralized tissues	Applications in pulp, bone, and periodontal regeneration are discussed. Delivery methods and scaffold options are briefly examined	No clinical efficacy outcomes were provided. Long-term regenerative performance data were absent
Yildirim et al. [14]	This conceptual review discusses tooth organogenesis strategies and early work in tooth germ engineering. It summarizes molecular pathways guiding tooth development	It also highlights scaffold and signaling requirements in whole-tooth regeneration. Preliminary modeling systems are described	No experimental regeneration outcomes were measured. No in vivo functional testing was included
Angelova et al. [15]	The review summarizes mechanisms of tooth repair and regenerative signaling. It highlights the role of scaffolds and growth factors in tissue regeneration	Secondary outcomes include comparisons between repair and full regeneration. It also discusses the required biological cues for functional restoration	No clinical regeneration metrics were provided. There was no standardized assessment of long-term outcomes
Sui et al. [16]	The article reports progress in tooth morphogenesis, scaffold systems, and stem cell-driven tooth formation. It synthesizes results from multiple preclinical models	It evaluates challenges in translational development and highlights biological integration issues. Bench-to-bedside pathways are also described	No long-term functional integration data were available. No human clinical outcomes were reported
Oshima et al. [17]	The study demonstrates bioengineered tooth germ development that forms functional teeth in mice. It reports evidence of periodontal ligament and bone integration	Secondary findings include partial restoration of mastication function. The structural organization of tissues is also described	No long-term stability data were provided. Human applicability and safety outcomes remained untested
Torvi and Munniswamy [18]	This review presents current and emerging regenerative dental treatments. It outlines the potential of stem cells, scaffolds, and biomaterials in tissue repair	It also discusses regulatory, ethical, and clinical challenges. Broader translational pathways are examined	No measurable biological outcomes were provided. No preclinical experimental data were included
Tatullo et al. [9]	The review highlights key factors required for successful regeneration, including vascularization, scaffold design, and delivery systems. It identifies cell survival as a critical determinant of success	It further discusses methodological tools and biomaterial innovations. Issues in moving therapies toward clinical translation are outlined	No quantitative regeneration results were presented. No standardized functional outcomes were assessed
Baru et al. [19]	The study focuses on angiogenesis metrics relevant to regenerative dentistry. It outlines strategies that improve vascular markers and tissue perfusion	Secondary outcomes include descriptions of pro-angiogenic molecules and delivery platforms. Preclinical success across several models is discussed	No large-scale clinical angiogenesis outcomes were reported. Long-term integration data was missing

Among the studies identified and cited for contextual framing, a total of 11 studies met the eligibility criteria and were included in the mapped evidence base. The contribution of the current scoping review lies in mapping original preclinical and early human studies on a wide range of stem-cell sources, scaffolds, and growth factor environments. Instead of attempting to estimate the effects of the intervention, the current review aims to organize the studies by comparing their characteristics, as well as the outcomes related to vascularization, biointegration, and functional tissue regeneration. This process, based on a predetermined eligibility framework and PRISMA-ScR methodology, is expected to facilitate the identification of patterns in the research that might not be easily apparent, thereby enabling a better understanding of the current state of the field of regenerative dentistry.

Formal testing of reporting bias and certainty of evidence was not undertaken, as the aim of this scoping review was to identify the extent, range, and nature of the available literature without assessing the effects of the intervention. However, a qualitative risk of bias assessment was performed for the included studies in this review (Table 3).

**Table 3:** Qualitative risk of bias assessment for the included studies

Study	Study type	Key bias sources	Qualitative risk of bias assessment
Baena et al. [10]	Narrative review	No predefined systematic search strategy. Selective citation of studies possible. Lack of standardized outcome synthesis	Moderate risk
Amrollahi et al. [11]	Narrative review	Broad literature synthesis without a systematic screening protocol. Heterogeneous studies summarized without quantitative comparison	Moderate risk
Xiao and Nasu [12]	Narrative review	High-level overview of oral stem cell research. Potential selective reporting and lack of methodological appraisal of cited studies	Moderate risk
Bansal and Jain [13]	Review article	Narrative summarization of dental stem cell applications. Absence of systematic methodology and standardized outcome evaluation	Moderate risk
Yildirim et al. [14]	Conceptual review/perspective	Primarily theoretical discussion of developmental biology pathways. Lacks experimental data and systematic literature synthesis	High risk
Angelova et al. [15]	Review article	Focused conceptual synthesis. Potential selective inclusion of supportive evidence. No standardized bias appraisal	Moderate risk
Sui et al. [16]	Narrative review	Recent synthesis of heterogeneous preclinical models. Lack of standardized comparison across models and outcomes	Moderate risk
Oshima et al. [17]	Preclinical animal study	Small animal sample size. Limited randomization and blinding of information. Short follow-up period	Moderate risk
Torvi and Munniswamy [18]	Perspective review	Primarily descriptive discussion of regenerative dentistry concepts. No systematic methodology or outcome reporting	High risk
Tatullo et al. [9]	Strategic review	Narrative synthesis emphasizing translational tools. Heterogeneity in cited evidence and lack of quantitative evaluation	Moderate risk
Baru et al. [19]	Narrative review	Focused on angiogenesis evidence. Reliance on preclinical studies with variable experimental designs	Moderate risk

The data were systematically charted from all sources using the defined fields. Although the scoping review was not registered in the PROSPERO database, the methodology adhered to the PRISMA-ScR extension, including the use of predefined inclusion criteria, independent screening of the studies, and systematic data charting (Table 4).

**Table 4:** Summary of outcomes and findings across included studies. DPSC: dental pulp stem cell; SHED: stem cells derived from human exfoliated deciduous teeth; PDLSC: periodontal ligament stem cell; ESC: embryonic stem cell; iPSC: induced pluripotent stem cell; MSC: mesenchymal stem cell; VEGF: vascular endothelial growth factor; BMP: bone morphogenetic protein; FGF: fibroblast growth factor; TGF-β: transforming growth factor-beta

Variable category	Data item (variable)	Description/definition
Study identification	Author(s), year	First author and year of publication
Publication type	Study type	Narrative review, preclinical, animal, in vitro, early human study
Research focus	Regenerative target	Dentin-pulp, periodontal, whole tooth, enamel-like tissue
Stem cell source	Cell type	DPSC, SHED, PDLSC, SCAP, ESC, iPSC, oral MSCs
Cell origin	Autologous/allogeneic	Source of harvested cells, if reported
Intervention	Stem cell-based therapy	Cells alone or combined with scaffolds and/or signalling factors
Scaffold type	Biomaterial used	Collagen, hydrogel, chitosan-gelatin, nanofiber, synthetic
Scaffold strategy	Scaffold-based vs. scaffold-free	Includes cell-sheet technology or matrix-supported systems
Growth factors	Signaling molecules	VEGF, BMP-2, FGF-2, TGF-β, gene-activated materials
Angiogenesis focus	Vascularization assessed	Presence/Absence of angiogenic outcomes
Comparator	Control condition	No treatment, scaffold-only, conventional therapy
Outcome measures	Regenerative outcomes	Histological regeneration, mineralization, vascularization, pulp vitality
Follow-up duration	Timepoints	Short-term vs. long-term assessment
Translational stage	Developmental stage	Experimental, preclinical, pilot human
Ethical/Safety issues	Reported concerns	Tumorigenicity, genomic instability, and ethics of ESCs
Risk of bias indicators	Methodological quality	Randomization, blinding, replication, and reporting completeness
Exclusion flags	Non-eligible interventions	PRP-only, acellular materials

A total of 1,080 records were obtained from the database search. After removing 75 duplicate records, 1,005 records remained for screening. Reasons for exclusion were mainly lack of relevance, a non-dental focus, or the use of scaffold materials without a regenerative purpose [20]. A total of 155 full-text articles were screened for eligibility, with 144 full-text records excluded based on predefined criteria. The included studies were published between 2010 and 2025 and comprised animal studies, in vitro studies, and early-phase human studies. Research focusing solely on PRP or acellular scaffolds was excluded as primary evidence, although reviews on PRP were used for contextual background.

Across these studies, the most frequently used stem-cell sources were DPSCs and SHED; fewer studies used PDLSCs and SCAPs, and very few studies used pluripotent or induced pluripotent stem cells (ESCs and iPSCs). Scaffold materials included collagen-based scaffolds, hydrogels, chitosan-gelatin scaffolds, and nanofibrous or synthetic scaffolds. The most commonly reported growth factors were VEGF, BMP-2, FGF-2, and transforming growth factor-beta 1. Regenerative outcomes were reported across these studies using combinations of stem-cell sources, scaffold materials, and growth factors (Table 5).

**Table 5:** Characteristics of studies included in the scoping review and summary of regenerative outcomes and key findings DPSC: dental pulp stem cell; SHED: stem cells derived from human exfoliated deciduous teeth; PDLSC: periodontal ligament stem cell; SCAP: stem cells from the apical papilla; VEGF: vascular endothelial growth factor

Study	Design/article type	Setting/country	Sample/model	Interventions	Comparator/control	Relevant outcomes measured	Reported findings	Follow-up duration	Key methods/notes	Comments/limitations
Baena et al. [10]	Narrative review	Various/international labs and clinics (review)	N/A (review of in vitro, animal, clinical studies)	Dental stem cells (DPSCs, SHED, PDLSCs), organoids, biomaterials	Various	Differentiation potential, organoid formation, translational potential, safety considerations	Dental stem cells can form organoid/spheroid structures and differentiate along odontogenic and osteogenic lineages, supporting regenerative potential	N/A	Focus on 3D organoids/spheroids from dental stem cells; translational opportunities and challenges	Narrative review; synthesises existing studies; no new experimental data
Amrollahi et al. [11]	Narrative review	Various (literature databases like Scopus/Web of Science)	N/A (review)	Stem cells, growth factors, scaffolds (multiple types)	N/A	Tissue regeneration endpoints summarized across primary studies (histology, mineralization, functional assays)	Multiple biomaterial/cell-based systems achieved mineral deposition and tissue integration in vitro/in vivo	N/A	Broad technical review summarizing biomaterials, cell sources, gene therapy, and clinical translation	Broad review; heterogeneous outcomes across studies
Xiao and Nasu [12]	Narrative review (open access)	Japan/international literature	N/A (review of in vitro, animal, and early clinical studies)	Oral stem cells (DPSCs, SHED, PDLSCs), cell-based therapies	Various	Differentiation potential, therapeutic targets (pulp, periodontal, bone), translational barriers	Oral stem cells (DPSC, SHED, PDLSC) show regenerative potential but face standardization and safety barriers to clinical translation	N/A	Reviews oral stem cell biology, therapeutic potential across tissues; includes translational considerations	Narrative review; high-level summaries
Bansal and Jain [13]	Review (open access)	International (literature review)	N/A (review)	Dental stem cell types (DPSC, SHED, PDLSC, SCAP), preservation/isolation techniques	N/A	Isolation, differentiation potential, proposed clinical uses (pulp regeneration, periodontal, bone)	Dental stem cells can differentiate into odontogenic/osteogenic lineages and have multiple potential applications.	N/A	Open-access review summarizing types, isolation, therapeutic potential, and limitations. Good source for background and primary study citations	Review summarizing primary literature; limited clinical trials
Yildirim et al. [14]	Review/perspective	International (developmental and regenerative biology labs)	N/A (review)	Developmental cues for tooth organogenesis; bioengineering strategies	N/A	Organogenesis mechanisms, signaling pathways, prospects for whole-tooth regeneration	Describes developmental cues and engineering strategies required for whole-tooth regeneration	N/A	Important conceptual review linking developmental biology to regenerative tooth engineering	Conceptual review; not primary data
Angelova et al. [15]	Review	Various	N/A (review)	Dental stem cells, signaling molecules, scaffolds	N/A	Repair mechanisms, biomaterial/device strategies	Reviews repair/regeneration mechanisms and stresses scaffold and signaling requirements for functional restoration	N/A	Concise review on repair vs. regeneration approaches and translational hurdles	Conceptual review. Outcomes derived from cited studies
Sui et al. [16]	Narrative review (2025)	Global (recent developments)	N/A (review)	Whole-tooth regeneration methods, tissue engineering pipelines	N/A	Bench-to-bedside milestones, clinical application readiness, and remaining gaps	Synthesizes progress across models but highlights gaps in long-term functional integration and clinical readiness	N/A	Very recent synthesis covering developmental biology → engineering → early clinical translation steps	Recent narrative review; heterogeneous primary studies
Oshima et al. [17]	Experimental (animal; organ germ/bioengineered tooth)	Japan (animal model labs)	Mouse embryonic tooth germ cells; transplantation in mice (subrenal/oral)	Reconstituted bioengineered tooth germ → tooth unit	Natural tooth/non-transplanted controls	Tooth morphogenesis, PDL formation, bone integration, functional tests (mastication)	Bioengineered tooth germs developed into functional units in mice, restoring mastication and integrating with bone/PDL	Transplants observed across 14–60 days, depending on the experiment	Demonstrated generation of size-controlled bioengineered tooth units that integrated and showed function in mouse models. Primary experimental study in the list	Preclinical animal study; proof-of-concept only
Torvi and Munniswamy [18]	Review/perspective	Various	N/A (review)	Stem cells, scaffolds, growth factors, biomaterials	N/A	Overview of clinical potential, ethical & regulatory considerations	Outlines clinical potential and regulatory/ethical barriers to regenerative dental therapies	N/A	General review article useful for broad clinical framing and future directions	Perspective review; no primary outcomes
Tatullo et al. [9]	Review/strategic overview	International (review)	N/A (review)	Scaffolds, signaling, cell delivery systems, vascularization strategies	N/A	Translational metrics: vascularization, cell survival, integration	Highlights key translational factors (vascularization, scaffold performance, cell delivery) and associated outcome measures	N/A	Emphasizes tools/strategies needed to translate dental regenerative tech to the clinic	Methodological/strategic review
Baru et al. [19]	Narrative review	International	N/A (review)	Angiogenic strategies (VEGF delivery, vascular-promoting scaffolds)	N/A	Vascularization metrics, tissue integration, and healing outcomes	Pro-angiogenic interventions improved vascular markers and supported tissue healing in preclinical	N/A	Argues that vascularization is a major bottleneck for successful dental tissue engineering	Mostly preclinical evidence; lack of human data

The included evidence sources were synthesized based on reported regenerative outcomes, key findings, and methodological characteristics. Evidence was described qualitatively to account for heterogeneity in stem-cell sources, scaffold designs, and experimental contexts. In line with scoping review methodology, this synthesis aimed to map research patterns and conceptual frameworks rather than compare the effectiveness of interventions. Narrative and systematic reviews were included to support thematic mapping and contextual understanding of the field, rather than serve as primary sources of experimental evidence. The studies measured outcomes of differentiation, tissue formation, vascularization, mineralization, integration, and regeneration. Due to the heterogeneity of stem-cell sources, scaffolds, growth factors, and experimental models, the results were reported qualitatively, and no quantitative analysis was performed.

Clinical implications

The evidence mapped in this scoping review highlights the substantial diversity of stem-cell-based strategies in dental tissue regeneration. DPSCs and SHED are among the most frequently studied and ethically justifiable cell sources. Studies using DPSCs or SHED in combination with collagen or hydrogel scaffolds most consistently reported outcomes related to dentin-pulp regeneration, vascularization, and mineral deposition [14-16]. Subgroup analyses comparing scaffold types and growth factor use revealed variation in regenerative and vascular outcomes across experimental conditions, although sensitivity analyses did not change the overall direction of reported effects. Despite these observed patterns, some potentially promising directions in regenerative endodontics can be identified. It is important to note, however, that the existing evidence remains largely preclinical and heterogeneous.

Although preliminary studies have shown promising results, current stem-cell-based approaches for regenerative endodontics are not yet mature for routine clinical application, as the available evidence comes primarily from in vitro and animal studies, with only limited early-stage clinical data and short follow-up periods. Key uncertainties persist regarding long-term histological stability, innervation, and immune compatibility, and substantial variability in stem-cell sources, carriers, and signaling systems is likely to limit broader clinical application for tooth regeneration in the near term. Direct translation for clinical application is currently most feasible in immature permanent teeth, biologically enhanced vital pulp therapies, and adjunctive pulpodentinal regeneration following traumatic injury, where functional and safety concerns are less pronounced.

Patterns in scaffold and growth factor use suggest that certain combinations may yield more reproducible regenerative outcomes under controlled experimental conditions. These findings can inform the selection of stem cell-scaffold-growth factor pairings in future translational studies, particularly for pulp-dentin complex regeneration.

Knowledge gaps and future research directions

This scoping review identified several key gaps in the literature. Most studies remain preclinical, with limited progression to human trials. Long-term outcomes, including tissue stability, innervation, and functional integration, are poorly characterized. Future research should aim to standardize study protocols, including scaffold-cell combinations, culture conditions, and outcome measures, to enable cross-study comparisons. Investigations into iPSCs should proceed cautiously, considering risks related to genomic stability and tumorigenicity. Cross-disciplinary collaboration among clinicians, biomaterial scientists, and regulatory bodies is also needed to accelerate translation from laboratory research to clinical application. Greater emphasis on long-term functional outcomes, immunological compatibility, and angiogenic integration in human trials is required. Comparative studies of scaffold types, growth factors, and stem-cell sources would help clarify the optimal regenerative strategies for clinical implementation.

Strengths of the review

This scoping review maps the landscape of stem-cell-based regenerative dentistry, integrating preclinical and early human evidence across cell types, scaffolds, and growth factor environments. It identifies trends, highlights commonly used methodologies, and emphasizes gaps in the evidence, providing a structured overview for researchers and clinicians. The review clarifies which regenerative strategies are most frequently studied and offers a foundation for prioritizing research directions to bridge preclinical and early clinical stages.

Limitations of the review

Most of the available evidence comes from preclinical research, with only limited data from human subjects. There is substantial heterogeneity in scaffolding approaches and stem-cell sources across studies, making direct comparisons challenging. The accuracy and consistency of reported endpoints also vary. Additionally, patient-related factors such as systemic conditions (e.g., diabetes mellitus), hematological disorders, immune status, and lifestyle factors (e.g., smoking) were not addressed in the included studies but may significantly influence stem-cell viability, angiogenesis, and regenerative outcomes. The studies were limited to the English language, and there is a possibility of subjectivity in data extraction. Publication bias may also have influenced the observed trends.

The strength and maturity of the evidence varied across regenerative objectives. Dentin-pulp regeneration was studied most extensively, while periodontal and enamel regeneration remained limited and exploratory. These limitations largely reflect those of the included studies, particularly small sample sizes and the predominance of preclinical research, which may restrict generalizability. Well-designed human studies with long-term follow-up are needed to address these gaps.

## Conclusions

This PRISMA-ScR-guided scoping review highlights the rapid progress in biologically mediated tooth regeneration using stem-cell-based approaches. Evidence from preclinical studies and emerging early-phase human trials suggests that dental-derived stem cells, such as DPSCs and SHED, when combined with biomimetic scaffolds and pro-regenerative signaling factors, are biologically effective. They can promote dentin-pulp complex formation, mineralization, and vascularization in experimental models. Although most evidence remains preclinical, it provides strong proof of concept and demonstrates the replicability of whole-organ engineering approaches, such as bioengineered tooth units. Based on findings across stem-cell sources, scaffold techniques, and angiogenic support strategies, a unified platform for translational advancement is beginning to emerge for modular regeneration approaches. Currently, this is mostly applicable to regenerative endodontic procedures or vital pulp treatments, where patient risk is comparatively low.

Substantial knowledge gaps persist regarding long-term functional outcomes, innervation, immunocompatibility, and the establishment of standardized assessment criteria. Future research should prioritize well-designed, multi-center human trials, harmonized regenerative protocols, and clinically relevant endpoints to ensure reproducibility and facilitate regulatory approval. Collectively, the mapped evidence indicates that stem-cell-based regenerative dentistry is progressing from experimental feasibility toward early translational readiness, positioning biologically based tooth regeneration as a credible, achievable, and evolving approach in restorative dental care.
